# A Systematic Review of Robotic Rehabilitation for Cognitive Training

**DOI:** 10.3389/frobt.2021.605715

**Published:** 2021-05-11

**Authors:** Fengpei Yuan, Elizabeth Klavon, Ziming Liu, Ruth Palan Lopez, Xiaopeng Zhao

**Affiliations:** ^1^Department of Mechanical, Aerospace, and Biomedical Engineering, University of Tennessee, Knoxville, Knoxville, TN, United States; ^2^School of Nursing, MGH Institute of Health Professions, Boston, MA, United States

**Keywords:** rehabilitation robotics, human-robot interaction, robot-assisted cognitive training, socially assistive robotics, multimodal interaction, cognitive disability

## Abstract

A large and increasing number of people around the world experience cognitive disability. Rehabilitation robotics has provided promising training and assistance approaches to mitigate cognitive deficits. In this article, we carried out a systematic review on recent developments in robot-assisted cognitive training. We included 99 articles in this work and described their applications, enabling technologies, experiments, and products. We also conducted a meta analysis on the articles that evaluated robot-assisted cognitive training protocol with primary end users (i.e., people with cognitive disability). We identified major limitations in current robotics rehabilitation for cognitive training, including the small sample size, non-standard measurement of training and uncontrollable factors. There are still multifaceted challenges in this field, including ethical issues, user-centered (or stakeholder-centered) design, the reliability, trust, and cost-effectiveness, personalization of the robot-assisted cognitive training system. Future research shall also take into consideration human-robot collaboration and social cognition to facilitate a natural human-robot interaction.

## 1. Introduction

It is estimated that ~15% of the world's population, over a billion people, experience some form of disability and a large proportion of this group specifically experience cognitive disability (WHO, 2011). The number of people with disabilities is increasing not only because of the growing aging population who have a higher risk of disability but also due to the global increase in chronic health conditions (Hajat and Stein, 2018). Individuals with cognitive disability, such as Alzheimer's disease (AD) or Autism spectrum disorder (ASD), may have a substantial limitation in their capacity for functional mental tasks, including conceptualizing, planning, sequencing thoughts and actions, remembering, interpreting subtle social cues, and manipulating numbers and symbols (LoPresti et al., 2008). This vulnerable population is usually associated with significant distress or disability in their social, occupational, or other important activities.

With recent advancements of robotics and information and communication technologies (ICTs), rehabilitation robots hold promise in augmenting human healthcare and in aiding exercise and therapy for people with cognitive disabilities. As an augmentation of human caregivers with respect to the substantial healthcare labor shortage and the high burden of caregiving, robots may provide care with high repeatability and without any complaints and fatigue (Taheri et al., 2015b). In a meta analysis comparing how people interacted with physical robots and virtual agents, Li (2015) showed that physically present robots were found to be more persuasive, perceived more positively, and result in better user performance compared to virtual agents. Furthermore, robots can facilitate social interaction, communication and positive mood to improve the performance and effectiveness of cognitive training (Siciliano and Khatib, 2016). For example, a recent study (Pino et al., 2020) showed that older adults with mild cognitive impairment (MCI) that received memory training through the humanoid social robot (NAO) achieved more visual gaze, less depression, and better therapeutic behavior. Physically embodied robots hold promise as accessible, effective tools for cognitive training and assistance in future.

There have been a few literature reviews on physical rehabilitation (Bertani et al., 2017; Kim et al., 2017; Morone et al., 2017; Veerbeek et al., 2017), or cognitive rehabilitation for specific user populations, such as children with ASD (Pennisi et al., 2016) and older adults (Mewborn et al., 2017). To the authors' best knowledge, this article presents the first systematic review that focuses on robotic rehabilitation for cognitive training. We present applications, enabling technologies, and products of robotics rehabilitation based on research papers research papers focusing on cognitive training. We also discuss several challenges to the development of robots for cognitive training and present future research directions.

## 2. Methods

### 2.1. Search Strategy

**Figure 1:**
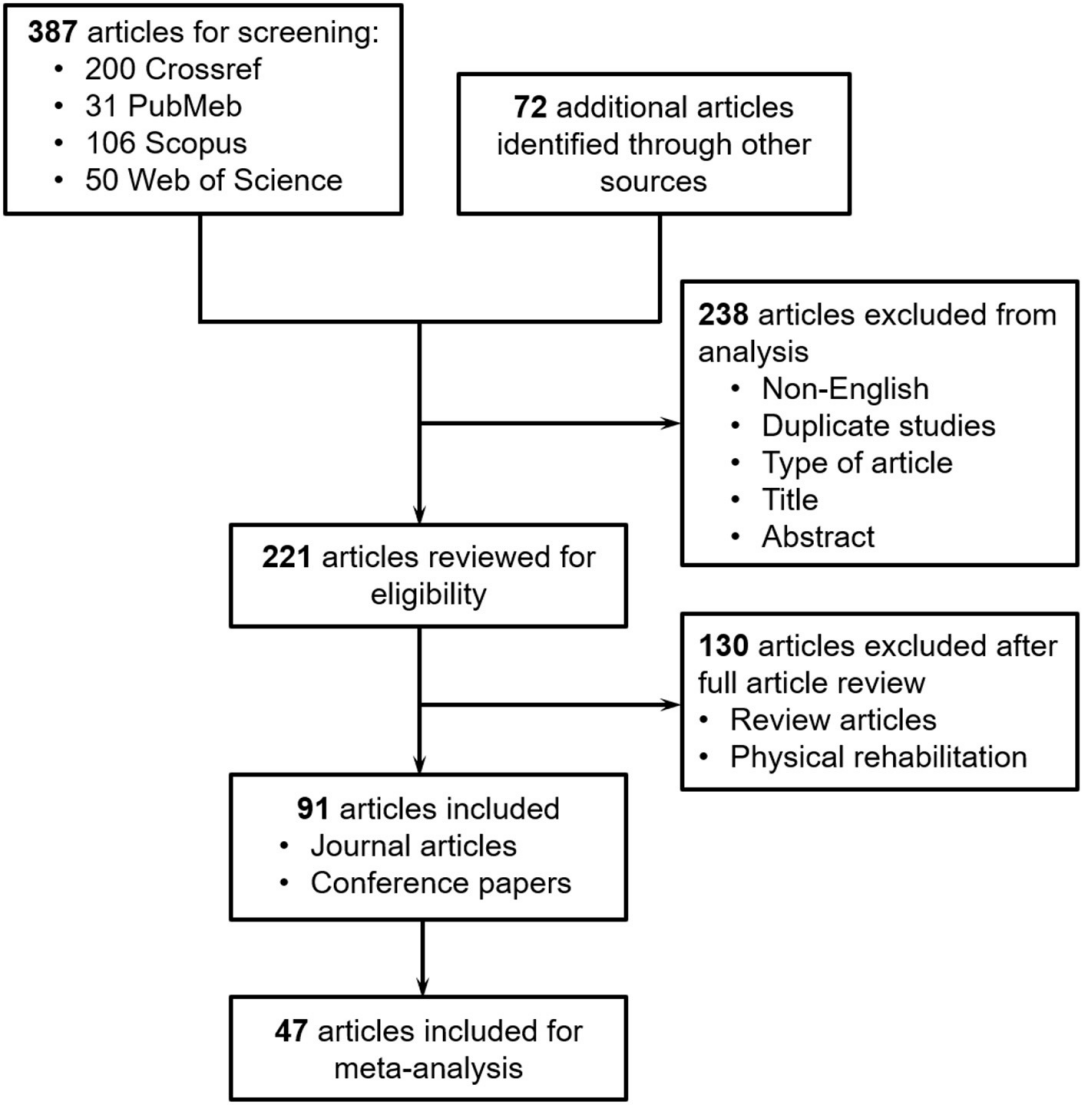
PRISMA flow diagram illustrating the exclusion criteria and stages of the systematic review.

We conducted a systematic review in the datasets of Google Scholar, Crossref, PubMed, Scopus, and Web of Science using the key words (“robot” OR “robots” OR “robotics” OR “robotic”) AND (“cognitive training” OR “cognitive rehabilitation” OR “cognitive therapy” OR “cognitive recovery” OR “cognitive restore”). The search was limited to the articles published between 2015 and December 14, 2020. The search in Google Scholar yielded 5,630 articles. Google Scholar ranks sources according to relevance, which takes into account the full text of each source as well as the source's author, the publication in which the source appeared and how often it has been cited in scholarly literature (University of Minnesota Libraries, 2021). We screened the titles of the first 500 articles and excluded the remaining results due to their low relevance. After the analysis of abstracts and written languages, 328 articles were further excluded due to duplication (i.e., exact copy of two works), non-English language, and/or not pertaining to the research topic. Then 172 full articles were reviewed for eligibility. The articles that were aimed for physical rehabilitation or review articles were excluded. Finally, 80 eligible articles were included for further analysis. As illustrated in the PRISMA flow diagram (see Figure 1), with the keywords we initially found 200, 31, 106, and 50 articles in the datasets of Crossref, PubMed, Scopus and Web of Science for screening, respectively. These articles were combined with the 80 additional articles from Google Scholar for further screening. After the analysis of titles, abstracts, written languages and types of article, 238 articles were excluded due to duplication, non-English language, non-eligible article types (e.g., book chapter, book, dataset, report, reference entry, editorial and systematic review), and/or not pertaining to the research topic. Then 229 full articles were reviewed for eligibility. The articles that were aimed for physical rehabilitation or review articles were excluded. Finally, 99 eligible articles were included in this systematic review, including journal articles and conference papers presented at conference, symposium and workshop. These papers included the contents of applications, user population, supporting technologies, experimental studies, and/or robot product(s). Moreover, 53 articles that included experimental study of robot-assisted cognitive training with primary end users (i.e., people with cognitive disability) were identified for further meta-analysis.

The literature include cognitive training robots in the forms of companion robots, social robots, assistive robots, social assisted robots, or service robots, which are collectively referred to as “cognitive training robots” in this systematic review. The literature employed different terminologies for cognitive training by rehabilitation robots, such as robot-enhanced therapy (David et al., 2018; Richardson et al., 2018), robot-assisted intervention (Scassellati et al., 2018), robot-assisted treatment (Taheri et al., 2015a), robot-assisted training (Tsiakas et al., 2018), robot-assisted therapy (Sandygulova et al., 2019), robot-mediated therapy (Begum et al., 2015; Huskens et al., 2015). Here, we do not distinguish between these different terms and instead adopt the term of “robot-assisted training” to represent all these different terms.

## 3. Results

### 3.1. Applications

The studies on robot-assisted cognitive training are categorized in terms of their applications and end users in Table 1. To date, the most researched application (36 out of 98 articles, as shown in Table 1) of robots in cognitive training is to improve individual social communication skills, which may include joint attention skills, imitation skills, turn-taking skills and other social interaction skills. For example, Kajopoulos et al. (2015) designed a robot-assisted training protocol based on response to joint attention for children with ASD. The training protocol used a modified attention cueing paradigm, where the robot's head direction cued children's spatial attention to a stimulus presented on one side of the robot. The children were engaged in a game that could be completed only through following the robot's head direction. To aid in the training of imitation skills in children with ASD, Taheri et al. (2020) proposed a reciprocal gross imitation human–robot interaction platform, in which ASD children are expected to imitate the robot body gestures, including a combination of arms, feet, neck, and torso movements. David et al. (2020) developed a robot-enhanced intervention on turn-taking abilities in ASD children. In their protocol, the robot provided instruction (e.g., “Now is my turn”) to the child, checked if the child moved the picture as instructed, and provided feedback (e.g., “Good job”) to the child if the child respected turns by staying with his or her hands still, without interrupting the robot. Robot-assisted cognitive training showed increased cognitive capabilities for people with limited social capabilities, such as children with ASD (Huijnen et al., 2016; Esteban et al., 2017; Marino et al., 2019) and people with dementia (Sung et al., 2015; Yu et al., 2015; Otaki and Otake, 2017). Due to cognitive impairment, individuals with dementia may also show deficits in social functioning, such as social withdrawal (Havins et al., 2012; Dickerson, 2015). In the pilot study by Sung et al. (2015) about robot-assisted therapy using socially assistive pet robot (PARO), institutionalized older adults showed significantly increased communication and interaction skills and activity participation after receiving 4-week robot-assisted therapy. Another robotic application is to provide intervention to enhance people's impaired cognitive function, such as memory (Paletta et al., 2018), attention (Lins et al., 2019) and concentration (Tleubayev et al., 2019), or reduce their negative psychophysiological feelings, such as stress (Aminuddin et al., 2016) and anxiety (Ab Aziz et al., 2015). Additionally, a few studies adopted the robots to facilitate learning and educational activities for people with cognitive disabilities, such as children with dyslexia (Andruseac et al., 2015) or Traumatic Brain Injury (TBI) (Barco Martelo and Fosch Villaronga, 2017).

**Table 1:** Type of robot-assisted cognitive training and end-user population.

**Application**	**User population**	**References**
Social communication skills	Children with ASD; Children with ADHD; Children with ID; People with dementia; People with CP; Older adults with social interaction problems	Begum et al., 2015; Conti et al., 2015; Costescu et al., 2015; Huskens et al., 2015; Kajopoulos et al., 2015; Miskam et al., 2015; Nunez et al., 2015; Sung et al., 2015; Taheri et al., 2015a, 2018, 2019, 2020; Yu et al., 2015; Zheng et al., 2015, 2016; Huijnen et al., 2016; Ozcana et al., 2016; Salvador et al., 2016; Santatiwongchai et al., 2016; Tariq et al., 2016; Wong and Zhong, 2016; Yun et al., 2016; Barco Martelo and Fosch Villaronga, 2017; Bharatharaj et al., 2017; Esteban et al., 2017; Otaki and Otake, 2017; Rudovic et al., 2017; Wood et al., 2017; David et al., 2018, 2020; Richardson et al., 2018; Scassellati et al., 2018; Ali et al., 2019; Marino et al., 2019; Melo et al., 2019; Sandygulova et al., 2019; Alnajjar et al., 2020
Memory	Children with CP; Older adults without CI; People with mild CI; People with dementia	Sonntag, 2015; Ahn et al., 2017; Garcia-Sanjuan et al., 2017; Tsardoulias et al., 2017; Paletta et al., 2018; Taranović et al., 2018a,b,c; Tsiakas et al., 2018; Nault et al., 2020; Pino et al., 2020
Concentration	Children with ASD; Children with ADHD	Tleubayev et al., 2019
Attention	Children with CP; Children with ID; People with mild CI; Older adults without CI; People with severe CI	Garcia-Sanjuan et al., 2017; Tsardoulias et al., 2017; D'Amico and Guastella, 2019; Lins et al., 2019
Visuo-spatial abilities	Children with impaired spatial abilities and WM	D'Amico and Guastella, 2019
Awareness	People with ABI	Yokota et al., 2019
Cognitive training (No specific cognitive function)	Children with ASD; People with mild CI; People with dementia; Older adults without CI; People with ID; People post-stroke	Kim et al., 2015, 2019; Kostavelis et al., 2015; Valent́ı Soler et al., 2015; Agrigoroaie et al., 2016; Coeckelbergh et al., 2016; Demetriadis et al., 2016; Lopez-Samaniego and Garcia-Zapirain, 2016; Salichs et al., 2016, 2018; Tsiakas et al., 2016; Abdollahi et al., 2017; Chu et al., 2017; Darragh et al., 2017; Khosla et al., 2017; Korchut et al., 2017; Shukla et al., 2017, 2019; Javed et al., 2018; Peleka et al., 2018; Rudovic et al., 2018; Andriella et al., 2019a,b, 2020; Law et al., 2019a,b; Pereira et al., 2019; Tokunaga et al., 2019; Calderita et al., 2020; Carros et al., 2020; Chen et al., 2020; Manca et al., 2020; Mois et al., 2020; Schüssler et al., 2020
Disruptive behavior problems	Children with DBD	Rabbitt et al., 2015
Anxiety	People with anxiety	Ab Aziz et al., 2015
Distress	Children with cancer	Alemi et al., 2016
Stress	People with stress	Aminuddin et al., 2016
Psychological healing	Not specified	Kohori et al., 2017
Hypnotherapy	Not specified	Alimardani and Hiraki, 2017
Education	Children with dyslexia; Children with ASD; Children with severe PD; Children with TBI; People with PMLD	Andruseac et al., 2015; Ioannou et al., 2015; Shukla et al., 2015; Taheri et al., 2016, 2019; Barco Martelo and Fosch Villaronga, 2017; Bharatharaj et al., 2017; van den Heuvel et al., 2017; Clabaugh et al., 2019
Vocational training	People with ASD; People with TBI	Bozgeyikli et al., 2015

*CI, Cognitive Impairment; ASD, Autism Spectrum Disorders; ADHD, Attention Deficit Hyperactivity Disorder; ID, Intellectual Disability; CP, Cerebral Palsy; WM, Working memory; ABI, Acquired Brain Injuries; PD, Physical Disability; TBI, Traumatic Brain Injury; PMLD, Profound and Multiple Learning Disabilities; DBD, Disruptive Behavior Disorders*.

### 3.2. Enabling Technologies

Recent development in robotics, ICTs, multimodal human-robot interaction, and artificial intelligence leads to significant process in robot-assisted cognitive training and rehabilitation. This section presents a summary on a few important enabling technologies that foster the advancement of robotic rehabilitation for cognitive training, including multimodal perception, multimodal feedback, gamification, virtual and augmented reality, and artificial intelligence.

#### 3.2.1. Design of Physical Appearance

A robot can have a human-like (Miskam et al., 2015; Peleka et al., 2018; Taheri et al., 2019), animal-like (Cao et al., 2015; Sung et al., 2015), or unfamiliar appearance (Scassellati et al., 2018). Besides the appearance, the size, softness, and comfort of the robot can also have an impact on users' perception, affection, cognitive load, and gaze following during interaction (Kohori et al., 2017), and thus the effectiveness of cognitive training. It remains unclear how users' perception is specifically affected by the robot's appearance. On the one hand, the human-like appearance was indicated to significantly positively affect users' perception of anthropomorphism, animacy, likeability, and intelligence toward robots, compared to a snowman-like appearance (Haring et al., 2016). On the other hand, increasing human-like appearance was found not to necessarily increase performance in human-robot interaction. For example, in the survey of expectation about the robots' appearance in robot-assisted ASD therapy, zoomorphic robots were indicated to be less ethically problematic than robots that looked too much like humans (Coeckelbergh et al., 2016). Some of their participants (i.e., parents, therapists, or teachers of children with ASD) worried about the possibility that the robot is perceived by the child as a friend, or that the robot looks too human-like. The relation between robots' human-like appearance and people's reaction to them may relate to the uncanny valley theory (Mori et al., 2012), which describes that people's familiarity (or affinity) with robots increases as the robots appear more human-like but when the robots are almost human, people's response would shift to revulsion. Tung (2016) observed an uncanny valley between the degree of anthropomorphism and children's attitudes toward the robots. In the review paper to study factors affecting social robot acceptability in older adults including people with dementia or cognitive impairment, Whelan et al. (2018) found that there is a lack of consensus regarding to the optimal appearance of social robots and that the uncanny valley concept varies between individuals and groups.

#### 3.2.2. Multimodal Sensing

Having a good understanding of a user's cognitive state, affective state and surrounding environment, which is termed as multimodal perception, is a prerequisite step for robots to provide cognitive training. Usually the concept of multimodal perception involves two stages, multimodal sensing and perception. Further details on these two techniques in previous publications are individually discussed in the following. Various sensors have been adopted to facilitate a robot to achieve multimodal sensing, based on system requirements, end-user population, cost-effectiveness, etc. Among different sensing technologies, visual and auditory sensing are the most popular modalities. We summarize the multiple modalities for sensing in the following aspects.

*1. Visual sensing*. During human-robot interaction, visual sensors are a very popular, useful and accessible channel for perception. The advancement of technologies, such as manufacturing and ICTs, enabled researchers to integrate small, high-resolution and affordable cameras into their rehabilitation robotic system. Some studies placed cameras in the environment along with the robot (Melo et al., 2019). Other studies included cameras in the robotic mechanical system. For example, in the social robot Pepper, there were 2D and 3D cameras attached to the head (Paletta et al., 2018). With computational approaches, such as computer vision, the robots analyzed the video/images from the cameras and recognized users' critical states, such as their environment, facial expression, body movements, and even emotion and attention (Paletta et al., 2018; Peleka et al., 2018; Rudovic et al., 2018; Mois et al., 2020), which led to a better perception of users (Johal et al., 2015).

*2. Auditory sensing*. Another popular modality adopted during robot-assisted cognitive training is auditory sensing. Researchers analyzed users' auditory signals in terms of the lexical field, tone and/or volume of voice for speech recognition, emotion detection, and speaker recognition in robots (Paletta et al., 2018; Peleka et al., 2018; Rudovic et al., 2018). Due to the natural and intuitive nature for users behind the auditory sensing, some end users, such as older adults preferred this sensing channel to the touch input during interaction with the robot (Zsiga et al., 2018).

*3. Physiological sensing*. Besides visual and auditory sensing, physiological modalities have been incorporated into the robotic system, in order to have a better understanding of users' states (e.g., affective states). Previous studies show that human-robot interaction may be enhanced using physiological signals, such as heart rate, blood pressure, breathing rate, and eye movement (Sonntag, 2015; Lopez-Samaniego and Garcia-Zapirain, 2016; Ozcana et al., 2016; Ahn et al., 2017; Alimardani and Hiraki, 2017). For example, Rudovic et al. (2018) employed wearable sensors to detect children's heart-rate, skin-conductance and body temperature during their robot-based therapy for children with ASD, to estimate children's levels of affective states and engagement. When studying robot-assisted training for children with ASD, Nunez et al. (2015) used a wearable device of electromyography (EMG) sensors to detect smiles from children's face.

*4. Neural sensing*. The inclusion of brain-imaging sensors provides capabilities to measure and/or monitor a user's brain activity and to understand the user's mental states (Ali et al., 2019). This was especially useful when considering the user's cognitive states, such as level of attention and task engagement (Alimardani and Hiraki, 2017; Tsiakas et al., 2018). Neural sensing may also be meaningful to users who have difficulty in expressing their intention and feeling because of their physical and/or cognitive limitations, such as the older adults with Alzheimer's disease. Currently, among all candidates of brain-imaging sensors, EEG and functional near-infrared spectroscopy (fNIRS) are two popular modalities due to their advantages of non-invasiveness, portability, and cost-effectiveness. For example, Lins et al. (2019) developed a robot-assisted therapy to stimulate the attention level of children with cerebral palsy and applied electroencephalogram (EEG) sensors to measure children's attention level during the therapy.

#### 3.2.3. Multimodal Feedback

After perceiving its user and environment, a robot shall entail multimodal feedback to interact (e.g., display its behaviors and feedback) with its user in a comfortable, readable, acceptable, and effective way (Melo et al., 2019). Multimodal feedback is particularly meaningful when the end users are unfamiliar with technologies or are limited in cognitive capabilities, such as older adults with dementia. Examples of feedback include voice, video, gesture, and physical appearance, all of which can affect users' perception of the robot during their interaction and thus the effectiveness of cognitive training/rehabilitation (Ab Aziz et al., 2015; Rabbitt et al., 2015). The following list shows popular modalities for robotic feedback during interaction with human users.

*1. Visual feedback*. One of the most widely-used overt feedback modalities is visual feedback or graphical user interface (GUI), displaying two-dimensional information. During robot-assisted cognitive training, visual feedback was delivered through an additional computer screen (Salvador et al., 2016; Taheri et al., 2019; Mois et al., 2020), or a touchscreen embodied in the robot (Paletta et al., 2018; Peleka et al., 2018). Some principles and/or issues on the design of GUI for robot-assisted training were suggested in previous studies. For example, a few studies recommended a larger screen and a simpler interface associated with each function choice for GUI to better facilitate visual feedback during cognitive training (Ahn et al., 2017).

*2. Auditory feedback*. Another widely used modality for feedback or interaction is auditory output, like speech during human-human interaction. This intuitive auditory communication can reduce the unfamiliarity with robotic technologies and increase the usability of system in the vulnerable population, such as the elderly population (Zsiga et al., 2018). Among previous studies, this feedback was delivered in one or combined form of beep (Nault et al., 2020), speech (Ab Aziz et al., 2015; Miskam et al., 2015; Peleka et al., 2018; Taheri et al., 2019), and music (Nunez et al., 2015). With auditory output, the robot provided daily communication and medication reminder, instructed cognitive training, and made emergent warning (e.g., short of battery) to the user (Orejana et al., 2015).

*3. Non-verbal feedback*. We refer to non-verbal feedback as all non-verbal communication cues by a robotic body, such as hand gestures and other body movements (Miskam et al., 2015; Taheri et al., 2019), eye gaze (Taheri et al., 2019), eye colors (Miskam et al., 2015; Taheri et al., 2019), and facial expression. Animation, similar to verbal language, makes a significant contribution to improving robot-assisted cognitive training. Moreover, the robot's animation can particularly introduce social interaction to a user, which is meaningful to individuals with impairments in social interaction skills.

*4. Haptic feedback*. Haptic feedback, by simulating the sense of touch and motion, may play important role during robot-assisted cognitive training due to the importance of touch in everyday life (Beckerle et al., 2017; Cangelosi and Invitto, 2017). Tactile feedback is one type of haptic feedback. During robot-assisted cognitive training, haptic feedback can also be introduced using vibration via wearable devices. For example, Nault et al. (2020) developed a socially assistive robot-facilitated memory game elaborated with audio and/or haptic feedback for older adults with cognitive decline. Although there was no significant difference in participants' game accuracy, preference, and performance in their system pilot study, the results provided insight into future improvements, such as increasing the strength of haptic feedback to increase the ease of being perceived and make the system more engaging. One notable robot, Paro, with the combination of soft, plush surface and additional encouraging haptic feedback (e.g., small vibration) creates a soothing presence. In a pilot study for institutionalized older adults by Sung et al. (2015), the communication and social skills of participants were improved by the robot-assisted therapy using Paro.

#### 3.2.4. Gamification

Recently, game technology is becoming a popular way to motivate, engage and appeal to users in cognitive tasks, since traditional cognitive tasks are usually effortful, frustrating, repetitive, and disengaging. Serious games and brain training games are a growing research field for cognitive training (Heins et al., 2017). Integration between gaming and robotic technologies has attracted increasing amount of interest in research and application, to further enhance users' engagement in cognitive training. A few types of relationships between robots and games have been developed in the literature. For example, a robot can lead or accompany users through the game for cognitive training by providing instructions on how to perform the task (Ioannou et al., 2015; Chu et al., 2017; Tsardoulias et al., 2017; Scassellati et al., 2018; Sandygulova et al., 2019; Taheri et al., 2019; Tleubayev et al., 2019; Nault et al., 2020) or playing a role of an active player in the game (Tariq et al., 2016; Melo et al., 2019). Additionally, a robot can provide various types of feedback (see details in section 3.2.3) to encourage users to engage in the game (Taheri et al., 2015a; Lopez-Samaniego and Garcia-Zapirain, 2016). Often, games associated with cognitive training can be integrated into the robotic systems through a GUI (Ahn et al., 2017; Paletta et al., 2018; Peleka et al., 2018).

#### 3.2.5. Virtual and Augmented Reality

Combining robot-assisted cognitive training with virtual reality (VR) and/or augmented reality (AR) techniques offers a cost-effective and efficient alternative to traditional training settings. The incorporation of VR/AR allows for replication of the tasks and environments in a more convenient and affordable way. Researchers also explored robotic cognitive training using mixed reality technology in cognitive training. For example, Sonntag (2015) presented an intelligent cognitive enhancement platform for people with dementia, where a mixed reality glass was used to deliver the storyboard (e.g., serious game for active memory training) to the user and a NAO robot served as a cognitive assistant for “daily routine.” Bozgeyikli et al. (2015) used virtual reality in a vocational rehabilitation system, which included six different modules, such as money management in a virtual grocery store, to provide vocational training for persons with ASD and TBI.

#### 3.2.6. Artificial Intelligence

Artificial Intelligence plays a significant role in the field of robot-assisted cognitive training/rehabilitation, including applications in multimodal perception and feedback, personalization, and adaptability (Ab Aziz et al., 2015; Rudovic et al., 2018). Given multimodal sensing, a successful multimodal perception further requires robots to integrate signals across multiple modalities of input sensors. To date, a great progress has been made thanks to the advancement of machine learning and deep learning. Multi-modal signals enable the robot with a good interpretation and understanding of its users, including their needs, intention, emotions, and surrounding environment (Paletta et al., 2018). Rudovic et al. (2018) implemented deep learning in a robot for ASD therapy to automatically estimate children's valence, arousal and engagement levels. Javed et al. (2018) utilized multimodal perception, including the analyses of children's motion, speech, and facial expression, to estimate children's emotional states. Using multiple feedback modalities may overload users with redundant information, increase task completion time, and reduce the efficiency of cognitive training (Taranović et al., 2018c). Additionally, users may favor certain modalities over others due to personal preference or cognitive disability. Taranović et al. (2018c) designed an experiment of adaptive modality selection (AMS) in robot-assisted sequential memory exercises and applied artificial intelligence to learn the strategy that selects the appropriate combination and amount of feedback modalities tailored to different situations (e.g., environments and users). An appropriate strategy is crucial to successful long-term robot-assisted cognitive intervention. Specifically, reinforcement learning, an area of machine learning, is a promising approach to adapt and personalize the intervention to each individual user as well as to optimize the performance of robot-assisted cognitive training, due to its capability of allowing a robot to learn from its experience of interaction with users (Sutton and Barto, 2018). For example, Tsiakas et al. (2016) used interactive reinforcement learning methods to facilitate the adaptive robot-assisted therapy, that is, adapt the task difficulty level and task duration to users with different skill levels (e.g., expert or novice user), in the context that users need to perform a set of cognitive or physical training tasks. Javed et al. (2018) developed a Hidden Markov model (HMM) in their adaptive framework for child-robot interaction, aiming to enable a child with ASD to engage in robot-assisted ASD therapy over long term. In their HMM, the states took into consideration a child's emotional state or mood, and the actions were the robot's behaviors or other audiovisual feedback. Clabaugh et al. (2019) utilized reinforcement learning to personalize instruction challenge levels and robot feedback based on each child's unique learning patterns for long-term in-home robot interventions. Although reinforcement learning may suffer the problem of sample inefficiency, the slowness of reinforcement learning can be overcome using techniques, such as episodic memory and meta-learning (Botvinick et al., 2019).

### 3.3. Experimental Studies

Many experimental studies have been conducted to evaluate the important properties of robotic rehabilitation, such as feasibility, safety, usability, performance, etc. On the one hand, exploratory studies including surveys and interviews with users (e.g., patients, caregivers, and therapists) have been conducted to inform the next stage of study (Rabbitt et al., 2015; Coeckelbergh et al., 2016; Salichs et al., 2016; Darragh et al., 2017; Kohori et al., 2017; Korchut et al., 2017; Law et al., 2019b). On the other hand, researchers have conducted experimental studies to verify and/or validate robot-assisted cognitive training systems. Table 2 shows a meta analysis for experimental studies, where the robot-assisted cognitive training was provided to primary end users (i.e., persons with cognitive disabilities). Up to date, majority of the experimental studies were conducted in a controlled lab setting, and only a few studies were conducted in an environment simulating daily activities in real world (Scassellati et al., 2018).

**Table 2:** Meta analysis on end-user experiments of robot-assisted cognitive training.

**References**	**Participants**	**Country-term**	**Study design**	**Outcomes (after training)**
Abdollahi et al. (2017)	6 (1M) seniors with mild dementia and/or depression, aged 63–86	USA; 4–6 weeks	One-on-one (robot vs. human) pilot study; Each individual had 24/7 access to robot.	Participants established rapport with the robot and greatly valued and enjoyed having the robot in their room. Subjects spent ~130 min per day interacting with the robot.
Agrigoroaie et al. (2016)	1 male with physical disability and cerebellar ataxia, aged 73; 1 female with arthritis aged 83	UK; One ~1-h session	Interaction with the robot in one partner care facility.	The residents' reactions were positive and they found the robot useful.
Alemi et al. (2016)	11 children with cancer, aged 9.5 ± 1.63	Iran; 18 days, 8 sessions	WOZ; Randomized into robot-assisted therapy group vs. psychotherapy control group	Children's stress, depression and anger were considerably alleviated during robot treatment. Significant differences were observed between two groups.
Ali et al. (2019)	12 (11M) children with ASD, aged 3.7–10.4	Pakistan; 6 months, 8 sessions for each intervention	Two different therapies of human-robot interaction, with and without inter-robot communication	Each participant showed improved eye contact duration over the experiments. In imitation module, participants actuated both robots almost equally in recurring experiments.
Alnajjar et al. (2020)	11 boys with ASD, aged 9.03 ± 2.56	UAE; one 5-min session in 1st week (pilot) and following 7 weeks with 1 session/week	Dynamic interaction scenario; Pilot study and long-term study	In long-term study, all 6 participants portrayed a trend of increasing attention scores. However, the therapist and system assessment trends were similar for most of the patients.
Begum et al. (2015)	3 (3M) persons with ASD, aged 13–19	USA; 6–10 days; 10–19 sessions; 2–4 min/session	WOZ	Metrics of skill execution and prompt dependency together created a highly informative picture of how well different participants performed. HRI metrics (Gaze, communication, and affect) were unable to measure the efficacy of the robot in achieving the goal of the therapy.
Bharatharaj et al. (2017)	9 children with ASD, aged 9.33 ± 3.39; 9 children's parents; 1 pediatrician; 1 psychologist	India; 5 consecutive days; Three 15-min sessions/day	Pilot study; WOZ; The robot was taught in the presence of children, who are expected to be curious by the robot and compete with the robot.	Results indicated that children with ASD appeared attracted and happy to interact with the parrot-inspired robot.
Chu et al. (2017)	139 (43M) seniors with dementia, aged 65–90	Australia; ≥5 years; Mostly 1 trial, 4–6 h/trial	Observational study in real life;	Social robots can improve diversion therapy service value to PwD through sensory enrichment, positive social engagement and entertainment.
Clabaugh et al. (2019)	17 children with ASD, aged 3–7	USA; 41 ± 5.92 days, encouraging 5 sessions/week, 10 games/session	In-home SAR intervention; Single-subject design for subjective measures	Each child participant was engaged with most intervention and showed improved targeted skills and long-term retention of intervention content. The robot system was reported useful and adaptable by families.
Conti et al. (2015)	3 (3M) children with ASD and ID, aged 11–12	Italy; One 9-min session	WOZ; Robot-mediated imitated game	Suggesting that the robot can be effectively integrated in the ASD therapies currently used.
Costescu et al. (2015)	40 children with TD, aged 5.4 ± 0.4; 41 children with ASD, aged 8.4 ± 2.2	Romania; Not specified	Counterbalanced; Each participant went through a robot condition and a human condition for reversal learning task.	Children with ASD were more engaged in the task and seemed to enjoy more in the robot condition vs. human condition. Their cognitive flexibility performance was generally similar in the robot and human conditions.
David et al. (2018)	5 (4M) children with ASD, aged 3–5	Romanian; 20 days, one 10-min session/day	Single-case alternative treatments design; Rapid alternation of 2 treatments; WOZ	A very consistent pattern across all types of sessions: using more cues (i.e., gaze orientation, pointing, and vocal instruction) for prompting JA increased children's performance.
David et al. (2020)	5 (3M) children with ASD, aged 3–5	Romania; 20 sessions, 1 session/day, 5–15 min/session	Single-case alternative treatments; Robot-enhanced treatment (RET) vs. standard human treatment (SHT); WOZ	Most children reached similar levels of performance on turn-taking skills across SHT and RET, meaning that children benefit to a similar extent from both interventions. The Robot partner seemed to be more interesting to ASD children than human partner.
Demetriadis et al. (2016)	45 (9M) persons with mild CI, age not specified	Greece; ~8 weeks, Once per week, 45–60 min/session	Randomized: intervention group with programming tasks vs. control group	Significantly improved post-test performance in “Test of Everyday Attention” in intervention group vs. control group.
D'Amico and Guastella (2019)	1 boy with impaired spatial abilities and WM, aged 15	Italy; 1 week, 6 activities, 30–60 min each activity	The boy followed the RE4BES protocol.	Improvement in 4 WM abilities, no improvements in short-term visual memory span, a worsening in word span
	1 boy with ID and severe difficulty on focused attention, aged 10	Italy; 1 month, 2 meetings/week	Single case quasi-experimental design	Significantly reduced problem behavior.
Garcia-Sanjuan et al. (2017)	40 (8M) seniors with no, mild and severe CI, aged 81.33 ± 8.48	Spain; 3 tasks, ~10–50 s/task	Usability study; Each user performed tasks individually	It is usable and engaging for users with no or mild CI. It is less usable for persons with severe CI, but triggering positive emotional reactions among them.
Huskens et al. (2015)	3 boys with ASD, aged 5–10; 3 healthy sibling aged 7–11	Netherlands; 3–5 sessions; 30 min/session;	Concurrent multiple baseline design across 3 child–sibling pairs; 3 pairs were randomly assigned to different baseline lengths of three, four, and five sessions.	No statistically significant changes in ASD children's collaborative behaviors.
Ioannou et al. (2015)	1 boy with high functioning ASD, aged 10	Cyprus; Four 20-min sessions	Single-case study; The boy played game with the robot and therapist	From session to session, the boy became more independent, initiating interaction with NAO, directing his gaze and expressing affective feelings.
Javed et al. (2018)	3 boys with ASD, aged 7–15; 3 (2M) neurotypical children, aged 4–9	USA; Activity time not specified	Preliminary study; Test vs. control group; 2-stage activity targeted at sensory processing skills	ASD children initiated more physical contact with the robot on average compared to neurotypical group. Children from both groups waved and smiled at the robot, and displayed imitation by attempting to emulate the robot's dance.
Kajopoulos et al. (2015)	7 (4M) children with ASD, aged 4–5	Singapore; 3 weeks, six 20-min sessions	3 phases: pre-test, robot training and post-test	Improved RJA skills after training. RJA skills were transferred from interaction with robot to with human experimenter.
Khosla et al. (2017)	115 seniors with dementia, aged 65–90	Australian; ≥1 trials; 4–6 h/trial;	Each trial involved 3 stages: introduction of robot, interaction with robot, and robot played games with users.	A statistically significant improvement in emotional, visual, and behavioral engagement of older people with social robots over the years. Their acceptance in the interaction with social robots was verified.
Kim et al. (2015)	48 seniors without CI, aged ≥60	South Korea; 12 weeks, 5 days/week, 90 min/day	Randomized: traditional CT vs. robot-assisted CT vs. without CT	Attenuation of age related cortical thinning in both CT groups. Less cortical thinning in the anterior cingulate cortices in robot group.
Kim et al. (2019)	48 seniors with mild CI, aged ≥60	South Korea; 4 weeks, 60 min/day	Single-blind RCT; Robot intervention group vs. control group	Greater improvement in attention in robot intervention group vs. control group.
Law et al. (2019a)	10 (4M) seniors with no or mild CI, aged 75–101; 2 experts in aged care	New Zealand; 1–3 sessions, ~60 min/session	Quantitative and qualitative design to gather users' and observers' feedback	Both users and experts believed the potential of robot-assisted cognitive game to improve cognition in people with MCI. Many functional issues with robot needed to improve.
Lins et al. (2019)	5 (3M) children with mild to moderate CP, aged 4–7	Brazil; 2 months, 2 sessions/week;	Group sessions; 3-phase game where children manipulated the robot	All children improved their performances on at least one level of difficulty for the exercise, with only two children failing to reach the third and last level of difficulty.
Lopez-Samaniego and Garcia-Zapirain (2016)	7 (3M) seniors with PI and CI, aged 78.0 ± 7.75	Spain; Once every 3 months, 25 min/session	All subjects participated the same cognitive and physical exercise.	Users were satisfied with the system usability (mean SUS score, 79.29).
Manca et al. (2020)	14 (5M) seniors with mild CI, aged 75.3 ± 4.5	Italy; 12 sessions over 1 month, 2 days/week	Randomized in terms of technology familiarity; robot- vs. tablet-assisted music game.	Participants in the tablet group provided more correct answers during game than the robot group. The robot was received with more enthusiasm by the older adults.
Marino et al. (2019)	14 (12M) children with ASD, aged 4–8	Italy; 10 sessions, twice a week, 90 min/session	RCT; Randomized in terms of gender; Robot-assisted intervention vs. control group; Group sessions	Substantial improvements in contextualized emotion recognition, comprehension and emotional perspective-taking through the use of human-assisted social robots.
Mois et al. (2020)	11 (3M) seniors with forgetfulness, aged 74.64 ± 6.02	USA; 4 weeks, 30-min session per week	WOZ	Engaging with the SAR improved participants' cognitive function across multiple domains
Otaki and Otake (2017)	6 seniors with coimagination, aged 73	Japan; 1 session; session duration not specified	WOZ	The robot could fulfill its role as a moderator, but the impression of robotic motion was not so good and the robot did not extend the topic by the question.
Pino et al. (2020)	21 (11M) seniors with mild CI, aged 73.45 ± 7.71	Italy; 8 weeks, weekly 90-min meeting	Group format; Training conditions robot- vs. human- assisted	Robot-assisted memory training increased patients' visual gaze and reinforced therapeutic behavior.
Rudovic et al. (2017)	36 (30M) children with ASD, aged 3–13	Japan and Serbia; One 25-min session	Exploratory analysis; WOZ; 2 Groups of Japan and Serbia	Statistically significant differences in engagement displayed in the two groups.
Salvador et al. (2016)	11 (9M) children with high-functioning ASD, aged 9.8 ± 2.9	USA; 5 weeks, 1 session/week	2 initial baseline sessions; 3 robot assisted intervention sessions.	There is correlation between reinforcer preference and age.
Sandygulova et al. (2019)	14 (12M) children with ASD and ADHD, aged 3–8	Kazakhstan; ≤ 6–15-min sessions	Iterative interaction design; 2 Phases; Design involving therapists, doctors and parents	Robot-assisted play had positive outcomes for most children.
Santatiwongchai et al. (2016)	6 (5M) children with ASD, aged 3–10	Thailand; ≤ 6 blocks of imaging matching game	Preliminary experiment; The robot as a medium for children with ASD and their parents in the game	Results varied among the children. Generally, response time and the number of incorrect answers decreased. Children often lost concentration during experiment.
Scassellati et al. (2018)	12 children with ASD, aged 9.02 ± 1.41	USA; 1 month, 30 min/day	Home-based intervention; Child-robot-caregiver interaction	The system maintained engagement over the 1-month deployment. Children showed improved JA skills with adults when not in the presence of the robot. Caregivers reported less prompting over time and overall increased communication.
Shukla et al. (2017)	30 (12 M) persons with ID, aged 45.24 ± 11.28; 6 caregivers, aged 38.6 ± 9.24	Spain; 2 days, 1 session/day, 10–20 min/session	Groups with robot-assisted cognitive stimulation vs. only caregiver	A significant reduction in caregiver workload in robot group. Disadvantages of robotic technical limitation.
Shukla et al. (2015)	6 (1M) persons with moderate to severe ID, aged 33–67	Spain; 3 months, 15–30 min/trial	Case study; 4 categories of participant-robot interactions	Participants showed 33 (out of 54) perfect responses. Irrespective of their mental condition all the participants were able to engage fully with the robot during interaction. All participants showed either a reduced or at-least same level of disability behavior during robot interaction trials comparing to normal situation behaviors.
Sung et al. (2015)	16 seniors with social interactions problems, aged ≥65	Taiwan; 4 weeks, two 30-min sessions/week	Robot assisted therapy in group session	Significantly improved communication and interaction skills (*z* = −2.94, *P*= 0.003) and activity participation (*z* = −2.66, *P*= 0.008) in participants after therapy.
Taheri et al. (2015a)	2 twin boys with ASD, aged 7	Iran; 6 weeks, two 30-min sessions/week	Individual and group sessions; Robot-Patient and Robot-Patient-Brother/Parent	Both participants showed greatly improved joint attention, pointing, and gaze shifting.
Taheri et al. (2018)	2 twin boys with ASD, aged 7	Iran; 6 weeks, two 30-min sessions/week	Single subject design using WOZ; Robot-Child or Robot-Child-Brother/Parent/Therapist interactions	The JA scores of both participants vs. treatment time showed linear shape of 0.3704 and 0.2589 (*p* = 0.02). A decrease in autistic and maladaptive behaviors in child with low-functioning ASD. The communication of both participants with each other improved.
Taheri et al. (2019)	4 boys with ASD, aged 6–7	Iran; 11 weeks (11 sessions), 20–30 min/session	Case study design; WOZ; pre-, post-, follow-up test	As a tool and facilitator, the robot was able to teach musical notes/rhythms to participants with high-functioning ASD. The severity of children's autism as well as the stress of the parents decreased somewhat during sessions. Noticeable improved social/cognitive skills in all participants.
Taheri et al. (2016)	4 boys with ASD, aged 6	Iran; 11 sessions, 20–30 min/session	Single subject design study; WOZ	All participants showed improvement in playing rhythm. The program affected positively on ASD severity, fine movement and communication skills.
Taheri et al. (2020)	20 (14M) children with ASD, aged 4.95 ± 2.01; 20 (10M) children with TD, aged 5.30 ± 1.95	Iran; Not specified	Counterbalance condition; Random order of robot-child interaction and human-child interaction; WOZ;	While the TD group showed a significantly better imitation performance than the ASD group, both ASD and TD groups performed better in the human-child mode than the robot-child mode.
Tariq et al. (2016)	3 (3M) children with ASD, aged 3.5–7	Pakistan; Four 15-min sessions	Exploratory study of robot-mediated play protocol	Increased execution, duration of target behaviors and social development (i.e., communicative competence, turn taking, and eye contact) of children with ASD with the robot-mediated play.
Tleubayev et al. (2019)	3 (2M) children with severe ASD and ADHD, aged 5–8	Kazakhstan; 21 days, 4–6 sessions on different days, ~15–20 min/session	Exploratory repeated-measures study	Sub 1: interested with the robot, and comprehension of tasks evolved throughout the experiment. Sub 2: Less noticeable dynamics in behavior. Sub 3: Significant improvement in eye contact with the robot and people outside the experiment.
Tokunaga et al. (2019)	21 (12M) healthy seniors, aged ≥65	Japan; 1 session, Session duration not specified	User study; Individual session.	Robot's appearance was acceptable; Participants had difficulty remembering story (correct rate ≤ 50%)
Valent́ı Soler et al. (2015)	101 (Phase1); 110 (Phase 2)	Spain; 3 months, 2 days/week, 30–40 min/session	Controlled clinical trial of parallel groups; Randomized by living units, stratified by dementia severity: CONTROL vs. PARO vs. NAO (Phase1) and CONTROL vs. PARO vs. and DOG (Phase2).	*Phase 1*: Improved apathy in patients in robot groups; Declined MMSE (but not sMMSE) scores in Patients in NAO; No significant changes between the robot groups. *Phase 2*: Increased QUALID scores in patients in PARO.
van den Heuvel et al. (2017)	17 children with severe physical disability, aged 2–8; 7 professionals	Netherlands; 2.5 months, 6 sessions, 2 individual sessions/week or 1 group session/week	Exploratory pilot study; WOZ; Children interacted with the robot in individual or group sessions.	A positive contribution of the robot in achieving therapy and educational goals. Sessions with robot were indicated as playful. The robot can contribute toward eliciting motivation, concentration, taking initiative and improving attention span of children.
Wong and Zhong (2016)	8 (6M) children with ASD, aged 5.3 ± 0.5	Singapore; 5 weeks, one 45-min session/week	Between conditions and within subjects design. Randomized to control condition and robot training condition	90% of children achieved some or all of individual pre-set aims. Significantly improved turn-taking skills and JA, and longer duration in eye contact engagement in children in robot condition.
Yun et al. (2016)	8 children with minimum competency level of age-appropriate cognitive skills, aged 3–5	South Korea; 8 sessions, 30–40 min/session	8 sessions were executed using iRobiQ and CARO equally; Child-therapist-robot interaction	Highest accuracy of 85.7% by robot in eye contact recognition; Gradually declined total eye contact rate during sessions. Progressively increased correct answer rate (≥72.25%) in reading emotions in participants.
Zheng et al. (2016)	6 boys with ASD, aged 2.8 ± 0.37	USA; 4 sessions across 32.5 days; Session 5 and 6 the same day	User study; 4 sessions of one-target interventions; 2 sessions to evaluate JA skills after 8 months	This autonomous robotic system was able to elicit improved one-target JA performance in young children with ASD over 8 months.
Zheng et al. (2015)	4 children with ASD, aged 3.83 ± 0.54; 6 children with TD, aged 3.61 ± 0.64	USA; Four 3-min sessions	User study; 2 human-administered sessions and 2 robot-administered sessions for each participant	The robotic system drew more attention from the ASD children and taught gestures more effectively compared to a human therapist.

*M, male; RCT, Randomized Control Trial; PI, Physical Impairment; CI, Cognitive Impairment; CT, Cognitive Training; WOZ, Wizard-of-OZ robot control; WM, Working memory; ID, Intellectual disability; JA, Joint Attention; RJA, Responding to JA; TD, Typically Developing; CP, Cerebral Palsy*.

#### 3.3.1. Study Design

Most experimental studies included three phases: pre-training assessment (i.e., baseline assessment), robot-assisted cognitive training, and post-training assessment (Kajopoulos et al., 2015; Kim et al., 2015; Sung et al., 2015; Yu et al., 2015; Alemi et al., 2016; Taheri et al., 2016, 2019; van den Heuvel et al., 2017; Scassellati et al., 2018; Marino et al., 2019). The effectiveness of robot-assisted training was evaluated by the comparison of pre- and post-training assessments using machine learning or statistical methods (Kim et al., 2015; Yu et al., 2015; Scassellati et al., 2018; Marino et al., 2019; Taheri et al., 2019).

Most studies adopted the group-based design where participants were randomly assigned to control or intervention groups (Kim et al., 2015; Sung et al., 2015; Yu et al., 2015; Marino et al., 2019). Some researchers employed single-case designs (or single-subject designs) to investigate the impact of social robots on cognitive training (Ioannou et al., 2015; Taheri et al., 2015a; David et al., 2018). For example, Ioannou et al. (2015) conducted the single-case study to explore the potential role of co-therapist of humanoid social robot, NAO, during autism therapy session with one child with ASD. In their study, there are four intervention sessions, and one follow-up, post-intervention therapy session to examine the effectiveness of the therapy with NAO.

Sample sizes vary dramatically in the literature (Ioannou et al., 2015; Kajopoulos et al., 2015; Kim et al., 2015; Sung et al., 2015; Chu et al., 2017; Khosla et al., 2017; Rudovic et al., 2018), where some studies were conducted with hundreds of participants whereas some studies included only a few participants. Challenges to recruitment included accessibility of participants and their caregivers, participants' disability, and ethical issues (e.g., privacy).

In terms of the intensity and duration of robot-assisted cognitive training, due to the variety of applications and end users, there was also a great variation in the total number of training sessions as well as the session duration. For example, with respect to one single training session, it took from about 20 min (Shukla et al., 2015; Tleubayev et al., 2019) to 90 min (Kim et al., 2015). Corresponding to the total cognitive training period, it varied from a few days (Bharatharaj et al., 2017) to more than 5 years (Chu et al., 2017).

#### 3.3.2. Evaluation

Researchers employed subjective and/or objective evaluation metrics to evaluate the performance of robots in cognitive training. Subjective measurement include qualitative observation, interviews and questionnaires. Objective measurements evaluate the performance from a behavioral or neurophysiological level.

*1. Observation*. During the experiments or recorded video, the experimenters or professional therapists observed and evaluated participants' behaviors, such as affective feelings, eye contact, communication, and other related interactions, based on their knowledge and experience (Begum et al., 2015; Conti et al., 2015; Costescu et al., 2015; Ioannou et al., 2015; Shukla et al., 2015; Taheri et al., 2015a, 2019; Yu et al., 2015; Tariq et al., 2016; Wong and Zhong, 2016; Yun et al., 2016; Zheng et al., 2016; Abdollahi et al., 2017; Chu et al., 2017; Garcia-Sanjuan et al., 2017; Khosla et al., 2017; Rudovic et al., 2017; David et al., 2018; Marino et al., 2019; Sandygulova et al., 2019; Tleubayev et al., 2019). This measurement was a very practical, dominant metric during the study of children with ASD. With the development of ICTs, some studies also applied customized software (instead of human effort) to evaluate user's behaviors, such as smiles and visual attention (Pino et al., 2020).

*2. Interview*. Interviews were conducted with the primary users (i.e., patients), their caregivers (e.g., parents and other family caregivers), and therapists, to learn about users' opinion and experience, and the performance of the robot-assisted cognitive training (Yu et al., 2015; Bharatharaj et al., 2017; Darragh et al., 2017; Paletta et al., 2018; Sandygulova et al., 2019; Taheri et al., 2019; Tleubayev et al., 2019). As stated in the book on user experience (UX) lifecycle by Hartson and Pyla (2018), user interview is a useful, practical technique to understand users' needs, design solutions, and evaluate UX, all of which are basic fundamental activities in UX lifecycle. Specifically, interviews can be applied to extract requirements of people with cognitive disability and/or their caregivers, to create the human-robot interaction design concepts, and to verify and refine human-robot interaction design for cognitive training. For example, in the case studies by Orejana et al. (2015), older adults with chronic health conditions in a rural community used a healthcare robot (iRobi) in their homes for at least 3 months. Then participants were interviewed to learn personal accounts of participants's experience. Through the interview, the authors found that more familiar games may be easier for older people to relate to and therefore increase users' confidence and that a larger screen would make the functions easier to see and use. The interview also revealed that older people sometimes have less dexterity so making the touchscreen less sensitive to long presses may remove accidental triggering of functions.

*3. Questionnaire*. Most studies utilized questionnaires to evaluate the performance of robot-assisted cognitive training. Researchers adopted questionnaire(s) based on their targeted performance, such as targeted user groups (e.g., patients, caregivers, or therapists), targeted cognitive capabilities (e.g., memory or anxiety), and research goals (e.g., users' perception of robot or effectiveness of robot). A few studies designed their own questionnaires according to their study (Tariq et al., 2016; Abdollahi et al., 2017; Ahn et al., 2017; Bharatharaj et al., 2017; Khosla et al., 2017; van den Heuvel et al., 2017; Scassellati et al., 2018; Lins et al., 2019; Tokunaga et al., 2019). Table 3 shows a list of common questionnaires in the literature.

**Table 3:** Questionnaires used to evaluate performance of robot-assisted cognitive training.

**Performance**	**Questionnaire**	**References**
Global functioning and disability	World Health Organization Disability; Assessment Schedule 2 (WHODAS 2.0); Functional Rating Scale for Symptoms of Dementia (FRSSD); Instrumental Activities of Daily Living (IADL)	Shukla et al., 2015; Demetriadis et al., 2016
Quality of life	SF-12 scale; WHOQOL-BREF; Quality of Life in Late-stage Dementia (QUALID)	Valent́ı Soler et al., 2015; Lopez-Samaniego and Garcia-Zapirain, 2016
Cognitive functions (or severity)	Mini-Mental State Exam (MMSE); Severe Mini-Mental State Exam (SMMSE); CNS Vital Signs; Cambridge Neuropsychological Test Automated Battery (CANTAB); Alzheimer's Disease Assessment Scale- cognitive subscale (ADAS-cog); Gilliam Autism Rating Scale (GARS); Global Deterioration Scale (GDS); Clinical Dementia Rating (CDR)	Kim et al., 2015, 2019; Valent́ı Soler et al., 2015; Demetriadis et al., 2016; Taheri et al., 2019; Mois et al., 2020
Autism Severity	Gilliam Autism Rating Scale (GARS)	Shukla et al., 2015; Taheri et al., 2015a, 2016, 2018
Memory decline	Memory Assessment Clinics-Questionnaire (MAC-Q)	Pino et al., 2020
Adaptive behaviors	AAMR Adaptive Behavior Scale: residential and community (ABS-RC: 2)	Shukla et al., 2015
Activity participation	Activity Participation Scale	Sung et al., 2015
Social communication skills	Assessment of Communication and Interaction Skills (ACIS-C); Autism Social Skills Profile (ASSP)	Sung et al., 2015; Taheri et al., 2019
Attention	The Godspeed questionnaire; Early Social Communication Scale (ESCS); Joint attention assessment of Bean and Eigsti; Test of Everyday Attention (TEA)	Kajopoulos et al., 2015; Demetriadis et al., 2016; Scassellati et al., 2018
Perceptions of robots	Robotic Social Attributes Scale (RoSAS)	Mois et al., 2020
Robot acceptance	Technology Acceptance Scale	Pereira et al., 2019; Mois et al., 2020
Robot usability	System Usability Scale (SUS)	Miskam et al., 2015; Lopez-Samaniego and Garcia-Zapirain, 2016; Nault et al., 2020; Pino et al., 2020
Robot's psychosocial impact	Psychosocial Impact of Assistive Devices Scales (PIADS)	Pino et al., 2020
Robot's neuropsychiatric impact	Neuropsychiatric Inventory (NPI) APADEM-NH Apathy Inventory (AI)	Valent́ı Soler et al., 2015; Demetriadis et al., 2016
Robot effectiveness	Individually Prioritized Problem Assessment (IPPA)	van den Heuvel et al., 2017
Robot satisfaction	Questionnaire for User Interaction Satisfaction (QUIS)	Lopez-Samaniego and Garcia-Zapirain, 2016
User's personality	Based on Big Five personality traits	Agrigoroaie et al., 2016
User's experience	Intrinsic Motivation Inventory (IMI)	Nunez et al., 2015
Perceived workload	NASA Task Load Index (NASA TLX)	Shukla et al., 2017; Mois et al., 2020; Nault et al., 2020
Anxiety	Multidimensional Anxiety Children Scale (MASC); Children's Depression Inventory (CDI); Hospital Anxiety and Depression Scale (HADS); State-Trait Anxiety Inventory (STAI-X)	Alemi et al., 2016; Pino et al., 2020
Depression	Children's Depression Inventory (CDI); HADS; Cornell Scale for Depression in Dementia (CSDD); Geriatric Depression Scale (GDS)	Yu et al., 2015; Alemi et al., 2016; Demetriadis et al., 2016; Pino et al., 2020
Anger	Children's Inventory of Anger (CIA)	Alemi et al., 2016
Affect	Positive and Negative Affect Schedule (PANAS)	Nunez et al., 2015; Aminuddin et al., 2016
Parenting stress	Parenting Stress Index-Short Form (PSI-SF)	Taheri et al., 2019
Caregiver burden	Zarit Burden Inventory (ZBI)	Yu et al., 2015

*4. Behavioral measurement*. From a behavioral perspective, researchers measured the number of correct/incorrect responses, response time, and/or time to complete the activity by participants to evaluate the performance of robot-assisted training (Bozgeyikli et al., 2015; Costescu et al., 2015; Ioannou et al., 2015; Kajopoulos et al., 2015; Salvador et al., 2016; Shukla et al., 2017; Lins et al., 2019; Nault et al., 2020).

*5. Neurophysiological measurement*. The advancement of brain-imaging technologies and deep learning enables researchers to assess the impact of cognitive training on cognitive capabilities from a neurophysiological perspective, using brain-imaging tools, such as EEG, fNIRS or functional magnetic resonance imaging (fMRI) (Ansado et al., 2020). Researchers also applied such tools to detect changes in the brain associated with participants' cognitive capability as metrics to evaluate the performance of robots in cognitive training (Kim et al., 2015; Alimardani and Hiraki, 2017).

### 3.4. Robot Products

The development of technologies, such as manufacturing and ICTs, has led to the generation of mass-product robots for research, education and therapeutic applications (Wood et al., 2017; Pandey and Gelin, 2018). Particularly in the field of cognitive training/rehabilitation, the developed robots are featured with capabilities, such as the aforementioned multimodal perception and multimodal feedback to support the human-robot interaction during cognitive training. Table 4 shows commonly used robot products as well as the important features to enable these robots to assist cognitive training. Their specific applications in cognitive training among previous studies, for example, assisting the intervention for memory and social communication skills, are listed in Table 1.

**Table 4:** Features of common robot products for cognitive training.

**Name**	**Physical appearance**	**Multimodal sensing**	**Multimodal feedback**	**Available in market**	**References**
NAO	Human-like	Cameras; Microphones; Touch sensors	Animation; Conversation	Yes	Begum et al., 2015; Conti et al., 2015; Huskens et al., 2015; Ioannou et al., 2015; Miskam et al., 2015; Shukla et al., 2015, 2017; Sonntag, 2015; Taheri et al., 2015a, 2016, 2018, 2019, 2020; Valent́ı Soler et al., 2015; Zheng et al., 2015, 2016; Alemi et al., 2016; Tariq et al., 2016; Rudovic et al., 2017; Tsardoulias et al., 2017; van den Heuvel et al., 2017; David et al., 2018, 2020; Tsiakas et al., 2018; Ali et al., 2019; Marino et al., 2019; Sandygulova et al., 2019; Tleubayev et al., 2019; Alnajjar et al., 2020; Pino et al., 2020
Pepper	Human-like	Cameras; Microphones; Touch sensors; Infrared sensors	Animation; GUI; Conversation; Eye color changing	Yes	Nunez et al., 2015; Paletta et al., 2018; Carros et al., 2020; Manca et al., 2020; Nault et al., 2020; Schüssler et al., 2020
KASPAR	Human-like	Visual; Touch sensors	Animation; Conversation; Facial expression	No	Huijnen et al., 2016; Wood et al., 2017
Paro	Animal-like	Auditory sensor; Touch sensor; Light sensor; Posture sensor	Animation; Sounds	Yes	Sung et al., 2015; Valent́ı Soler et al., 2015; Yu et al., 2015; Aminuddin et al., 2016
Probo	Animal-like	Cameras; Microphones; Touch sensors	Animation; GUI; Conversation; Facial expression	No	Cao et al., 2015
CuDDler	Animal-like	Camera; Microphones	Animation; Sounds	No	Kajopoulos et al., 2015; Wong and Zhong, 2016
iRobiQ	Human-like	Camera; Microphone; Touch sensors	Animation; GUI; Conversation; Facial expression	Yes	Yun et al., 2016; Ahn et al., 2017
Silbot	Human-like	Camera; Microphones	Animation; GUI; Conversation	Yes	Kim et al., 2015; Law et al., 2019b
Mero	Human-like	Cameras; Microphone	Animation; GUI; Conversation; Facial Expression	No	Kim et al., 2015
Lego robot	Not applicable (Building bricks)	Changeable, Color sensor; Touch sensors; Infrared sensor	Changeable, Auditory; Tablet; Animation	Yes	Andruseac et al., 2015; Demetriadis et al., 2016; Lopez-Samaniego and Garcia-Zapirain, 2016; Garcia-Sanjuan et al., 2017; D'Amico and Guastella, 2019; Lins et al., 2019
RAMCIP	Human-like	Camera; Microphone; Laser scanners	GUI; Conversation; Facial expression	No	Kostavelis et al., 2015; Peleka et al., 2018
Jibo	Unfamiliar	Cameras; Microphones	GUI; Communication; Spin in 360°	Yes	Scassellati et al., 2018
Vän Robotics	Human-like	Camera	Animation; Communication	Yes	Mois et al., 2020
RoboKind	Human-like	Cameras; Microphones; Touch sensors	Animation; Conversation; Facial expressions	Yes	Taheri et al., 2015a, 2018; Salvador et al., 2016
Keepon	Snowman-like	Cameras; Microphone; Touch sensors	Animation; Sounds	Yes	Costescu et al., 2015
CARO	Human-like	Cameras; Microphones; Touch sensors	GUI; Eye emotional expressions	No	Yun et al., 2016
Kompaï	Human-like	Cameras; microphones	GUI; Communication	Yes	Agrigoroaie et al., 2016
InO-Bot	Turtle-like	Proximity sensors; Line follower sensors	Light (LED); Auditory	Yes	D'Amico and Guastella, 2019

## 4. Discussions

### 4.1. Limitations

#### 4.1.1. Sample Size

Probably, the most common challenge faced by researchers of cognitive training is a small size of participants. This imposes the generalization and reliability of experimental results in question. The limitation of small sample size was caused by the small number of participants or the limited number of available robots for experiments (Kajopoulos et al., 2015; Shukla et al., 2015; Zheng et al., 2015; Tariq et al., 2016; Bharatharaj et al., 2017; Darragh et al., 2017; Garcia-Sanjuan et al., 2017; Tsardoulias et al., 2017; Lins et al., 2019; Marino et al., 2019; Tleubayev et al., 2019). Typically, a research lab has only a few robots. This would be particularly challenging to experimental studies that require multiple sessions for each individual user. In this case, within the same study period, these studies have to control the number of participants to a small number. The recruitment of participants was influenced by the accessibility to participants during the whole study and sometimes the problems associated with their caregivers (Alemi et al., 2016; Taheri et al., 2019). For example, in the study (Alemi et al., 2016) of exploring effect of utilizing a robot NAO as a therapy-assistive tool to deal with pediatric distress among children with cancer. In terms of the small sample size, researchers mentioned that due to the novelty of their project and scant number of systematical psychological interventions for patients with cancer or other refractory illness in Iranian hospitals, it was difficult to persuade children's parents to join their study. Also, they mentioned that it was hard for parents to bring their kids to intervention sessions on a regular basis. Moreover, the issue of small sample size also means that the participants in some studies were not general and representative, in terms of factors, such as their severity of cognitive disability, their age and gender (Begum et al., 2015; Yu et al., 2015; Chu et al., 2017).

#### 4.1.2. Measurement of Training Effectiveness

Another impeding factor in studies of robot-assisted cognitive training is the evaluation of its training effectiveness, which can relate to choosing tools for specific evaluation metrics, identifying relevant evaluation metrics, or designing experiments to facilitate evaluation. In terms of the evaluation metrics, many studies adopted subjective evaluations, which could be biased and inaccurate. To the authors' best knowledge, there is no standardized questionnaire to evaluate robot-assisted cognitive training. As shown in Table 3, multiple different questionnaires were applied to evaluate the same target (e.g., robot acceptance), which makes it difficult to compare the performance of robot-assisted cognitive training between different studies (Bharatharaj et al., 2017). Often, assessment metrics focus on the impact on the specifically trained cognitive capability, ignoring the potential transfer to other cognitive skills (Zheng et al., 2016) and the long-term performance (Richardson et al., 2018). Moreover, evaluations were frequently conducted for the robot-assisted cognitive training in controlled laboratory settings. The real-world environments are usually noisy and dynamic, which brings greater challenges for a reliable, robust user-robot interaction and a good user experience of the robot (Salem et al., 2015; Trovato et al., 2015).

Additionally, the effectiveness of robot-assisted cognitive training may be impacted by users' perceived interaction with the robot. On the one hand, some studies (Lopez-Samaniego and Garcia-Zapirain, 2016; Pereira et al., 2019; Mois et al., 2020) have evaluated the acceptance, satisfaction and perception of robots for cognitive training. On the other hand, many studies (Kim et al., 2015; Shukla et al., 2015; Demetriadis et al., 2016; Pino et al., 2020) have evaluated effectiveness of robot-assisted cognitive training on participant's cognitive capabilities. However, it is rarely addressed in the literature how acceptance and perception of the robot affects the effectiveness of cognitive training. Moreover, as shown in Table 2, some studies presented the results of robot-assisted training without comparing to the effectiveness of human-assisted training. For more rigorous evaluation of the effectiveness of a robot-assisted cognitive training approach, it is recommended to compare against human-assisted training and other existing approaches.

#### 4.1.3. Uncontrollable Factors

There always exist uncontrollable factors during the study of robot-assisted cognitive training/rehabilitation. The problem is more noteworthy for multiple-session studies since researchers cannot control participants' daily and social activities outside of the laboratory setting. The topic of uncontrollable factors is relatively less studied. In a study on using a social robot to teach music to children with autism, Taheri et al. (2019) pointed out some improvements observed in music education and/or social skills are attributable to other interventions or education the participants may be receiving. When investigating the influence of robot-assisted training on cortical thickness in the brains of elderly participants, Kim et al. (2015) recognized uncontrollable factors due to participants' daily cognitive activity at home, such as using computers or reading books.

### 4.2. Challenges and Future Development

#### 4.2.1. Ethical Challenges

During the development of robots for cognitive training/rehabilitation, there are some ethical issues with respect to human dignity, safety, legality, and social factors to be considered. For example, during robot-assisted cognitive training, the interaction between the user and the robot happens at both the cognitive (dominant) and physical level (Villaronga, 2016). There could be the issue of perceived safety, or cognitive harm. For example, the user may perceive the robot unsafe or scary (Salem et al., 2015; Coeckelbergh et al., 2016). In the study (Shim and Arkin, 2016) exploring the influence of robot deceptive behavior on human-robot interaction, a robot NAO deceptively showed positive feedback to participants' incorrect answers in a motor-cognition dual task. The self-report results revealed that the robot's deceptive feedback positively affected a human's frustration level and task engagement. Even though a robot's deceptive action may lead to positive outcome, Shim and Arkin emphasized that the ethical implications of the robot deception, including those regarding motives for deception, should always be discussed and validated prior to its application. Another arising ethical issue is how responsibility can be allocated, or distribution of responsibility (Loh, 2019; Müller, 2020). For example, if a robot acts during cognitive training, will the robot itself, designers or users be responsible, liable or accountable for the robot's actions? We should also pay close attention to ethical issues, such as the affective attachments, dependency on the robot, safety and privacy protection of users' information, and transparency in the use of algorithms in robotic systems (Kostavelis et al., 2015; Casey et al., 2016; Richardson et al., 2018; Fiske et al., 2019). Similarly, designers should accommodate the design of robot to these ethical considerations (Ozcana et al., 2016). To ensure the perceived safety, researchers need to always take end users' perception into account, which can be known through questionnaires and interviews with them (and their caregivers and therapists if needed), and their behavioral and neurophysiological activities. The tendency for humans to form attachments to anthropomorphized robots should be carefully considered during design (Riek and Howard, 2014; Riek, 2016). Moreover, for fear that the robot could replace human health care from both patients and the professional caregivers, it should be emphasized that the rehabilitation robots are developed with the aim of supplementing human caregivers, rather than replacing them (Doraiswamy et al., 2019).

#### 4.2.2. User-Centered Design

The goal of robotic cognitive rehabilitation is to provide cost-effective cognitive training to vulnerable people with cognitive disabilities, which can supplement their caregivers and/or therapists (Doraiswamy et al., 2019). Therefore, we encourage the idea of user-driven, instead of technology-driven, robot design and development (Rehm et al., 2016). Emphasis should be given to the primary users (i.e., patients) of the robots and other key stakeholders (e.g., caregivers, therapists, and doctors) to design and shape this robot, including requirement analysis, robot development and evaluation with different stakeholders (Casey et al., 2016; Gerling et al., 2016; Leong and Johnston, 2016; Rehm et al., 2016; Salichs et al., 2016; Barco Martelo and Fosch Villaronga, 2017; Riek, 2017). It is also important to pay attention to potential technical difficulties for vulnerable populations, such as the elderly and children with ASD (Orejana et al., 2015) and the social and contextual environment that the robot will be applied to (Jones et al., 2015). More standardized, unbiased benchmarks and metrics need to be developed for different stakeholders to evaluate the performance of robots from their perspectives. While it is necessary to start pilot studies with healthy participants, it is crucial to relate the developed systems to patients with cognitive impairment at home settings.

Furthermore, robot development is a multidisciplinary study which requires knowledge from multiples fields, such as social cognitive science, engineering, psychology, and health care, such as ASD and dementia. Enhanced collaborations among these fields are needed to improve future technology in robotic rehabilitation.

#### 4.2.3. Reliability, Trust, Transferability, and Cost-Effectiveness

The reliability of the robotic system ensures that the robot can consistently work in noisy, dynamic real-world environment. This makes a significant contribution to a user's confidence and increases positive perception of the robot (Wood et al., 2017). Mistakes made by robots during interaction can cause a loss of trust in human users. More work on human-robot interaction is needed to implement a reliable and robust robot to assist cognitive training. This may cover multimodal sensing technologies, artificial intelligence, and modeling. On the other hand, we need to take into consideration how to effectively restore trust to the robot in case that mistakes are made by the robot during interaction with the user. This may involve how and when the robot apologizes for its mistake for users. Robinette et al. (2015) found that timing is a key to repair robot trust, suggesting that the robot should wait and address the mistake next time a potential trust decision occurs rather than addressing the mistake immediately.

Currently, most studies focus on specific cognitive training tasks and environments, which means that the robot cannot assist in other cognitive tasks. Here, we encourage the implementation of a transferable mechanism in robots for cognitive training. In other words, we should enable more powerful learning algorithms in the robot so that the robot can learn and adapt to more new cognitive training (Andriella et al., 2019a). Researchers should also take cost-effectiveness into account during the design of robot (Wood et al., 2017). From the commercial perspective, cost-effectiveness is considered beyond the purchase, maintenance, and training costs for the system (Riek, 2017). Furthermore, from the perspective of time and effort of users, more work is needed to find out the optimal robot-assisted cognitive training strategy (e.g., the frequency and duration of each cognitive session). Therefore, we encourage future studies clearly state the used training strategies, making it easier for the community to compare different strategies.

#### 4.2.4. Personalization

There is no “one-size-fits-all” in health care. To provide a successful cognitive training, the robot needs to be personalized and adaptive in three levels. Firstly, personalization requires the robot to provide appropriate cognitive training and feedback to meet the specific need of groups with different cognitive disabilities (e.g., people with ASD, people with ADRD). Secondly, the robots need to adapt to the diversity existing in the population as well as tailor to each individual user's severity of cognitive impairment, cultural and gender-dependent difference, personality and preference (Kostavelis et al., 2015; Javed et al., 2016; Riek, 2016; Darragh et al., 2017; Rudovic et al., 2017; Richardson et al., 2018; Sandygulova et al., 2019). For example, children with ASD, have a wide range of behavioral, social, and learning difficulties. And each individual may have a different preference to robot's gender and modalities of feedback (Sandygulova and O'Hare, 2015; Nault et al., 2020). As a result, we expect that a personalized robot would provide various cognitive training, e.g., a variety of games and adjustable voice, for diverse individual needs and requirements to keep the user engaged and focused over long term (Scassellati et al., 2018; Tleubayev et al., 2019). Furthermore, rehabilitation robots should adapt to individually time-changing characters, such as cognitive impairment, task engagement, and even personalities (Agrigoroaie and Tapus, 2016; Tsiakas et al., 2016, 2018). For example, the robot should adjust the cognitive training and feedback if the user feels bored, too difficult, or too easy (Lins et al., 2019). Machine learning methods should also take into consideration of personalization. Existing methods, such as interactive reinforcement learning (IRL) or incremental learning (Castellini, 2016) provide good examples, where one block module is used to specifically model each user's information, such as patient's name, hobbies and personalities related to cognitive training (Salichs et al., 2016). IRL is a variation of reinforcement learning that studies how a human can be included in the agent learning process. Human input play the role of feedback (i.e., reinforcement signal after the selected action) or guidance (i.e., actions to directly intervene/correct current strategy). IRL can also be utilized to enable adaptation and personalization during robot-assisted cognitive training. For example, Tsiakas et al. (2016) proposed an adaptive robot assisted cognitive therapy using IRL, where the primary user feedback input (e.g., engagement levels) were considered as a personalization factor and the guidance input from professional therapist were considered as a safety factor. Their simulation results showed that IRL improved the applied policy and led to a faster convergence to optimal policy. Castellini (2016) proposed an incremental learning model to enforce a true, endless adaptation of the robot to the subject and environment as well as improve the stability and reliability of robot's control. Incremental learning enables an adaptive robot system to update its own model whenever it is required, new information is available, or the prediction is deemed no longer reliable.

#### 4.2.5. Human-Robot Collaboration

Future rehabilitation robots should not only be autonomous but also be collaborative (or co-operative) (Huijnen et al., 2016; Weiss et al., 2017). From the perspective of collaboration between the robot and the primary end users (i.e., people with cognitive disability), there is evidence indicating that a fully autonomous robotic system is not the best option for interaction with the vulnerable population (Peca et al., 2016). Instead, a semi-autonomous robot is a more suitable solution (Wood et al., 2017). With the highest-level goal of enhancing user's cognitive capabilities, the robot should “care” about the user's situation, take compensatory reaction as a teammate, engage the user and train/stimulate the user's cognitive capabilities as best as possible. The capability of collaboration may also help to avoid the user's feeling of redundancy and increase their feeling of self-autonomy and long-term engagement in cognitive training (Orejana et al., 2015). The robot should have a good perception of the user's changing situations and an intelligent strategy to engage the user. On the other hand, from the perspective of collaboration among robots, users, and their caregivers (and therapists), more future work is needed to solve the shared control issue. Researchers need to figure out strategies for robots to render the caregivers' and therapists' tasks easier as an assistive tool for cognitive training, instead of totally replacing them (Kostavelis et al., 2015; Coeckelbergh et al., 2016). A distribution between autonomy of robots and teleoperation by caregivers/therapists is needed to support the collaboration of robots for cognitive training.

#### 4.2.6. Social Cognition

The knowledge gained in human-human interaction can be applied to foster human-robot interaction and to obtain critical insights for optimizing social encounters between humans and robots (Henschel et al., 2020). Marchesi et al. (2019) conducted a questionnaire study to investigate whether people adopt intentional stance (Dennett, 1989) toward a humanoid robot, iCub. Their results showed that it is possible to sometimes induce adoption of the intentional stance toward humanoid robots. Additionally, non-invasive neuroimaging techniques (e.g., fMRI) in neuroscience enable the possibility of probing social cognitive processing in human brain during interaction with robots. For example, Rauchbauer et al. (2019) observed that neural markers of mentalizing and social motivation were significantly more activated during human-human interaction than human-robot interaction. Klapper et al. (2014) showed that human brain activity within the theory-of-mind network (Saxe and Wexler, 2005; Price, 2012; Koster-Hale and Saxe, 2013) was reduced when interacting with agents in a form of low human animacy (i.e., physical appearance) compared to high human animacy. These issues become important when we adopt robots for cognitive training for people with cognitive dysfunction (Frith and Frith, 1999; Chevallier et al., 2012), as they underline the substantial contrast between human-human and human-agent interactions. Additionally, the advanced non-invasive, portable, and cost-effective neuroimaging techniques (e.g., EEG and fNIRS) hold the promise of evaluating human-robot interaction from controlled laboratory setting to real-world setting. Herein, we encourage to leverage human neuroscience to facilitate the development of robots for cognitive training, such as understanding the effects of robot-assisted cognitive training and learning the extent and contexts at which it can be beneficial from neurophysiological perspective.

#### 4.2.7. Natural Human-Robot Interaction

Similar to human-human interaction during cognitive training by human therapists, robots need to be able to interact with users naturally in robotic rehabilitation. This includes having a good understanding of user's emotions (e.g., happiness, shame, engagement), intentions and personality (Pettinati and Arkin, 2015; Rahbar et al., 2015; Vaufreydaz et al., 2016; Rudovic et al., 2018), being able to provide an emotional response when being shared with personal information (de Graaf et al., 2015; Chumkamon et al., 2016), talking day-by-day more to the user on various topics like hobbies, and dealing with novel events (Dragone et al., 2015; Kostavelis et al., 2015; Adam et al., 2016; Ozcana et al., 2016). These natural user-robot interactions require powerful perception, reasoning, acting and learning modules in robots, or in other words, cognitive and social-emotional capabilities. However, from the perception perspective, understanding users' intentions and emotions is still a great challenge for robots (Matarić, 2017). Robots need to interpret multimodal signals (e.g., facial expression, gestures, voice, and speech) simultaneously to understand users' covert intentions and emotions. Similarly, more work is needed for the multimodal feedback in the future. To maximize the benefits of physically present robots and facilitate both the short- and long-term human-robot interaction for cognitive training, we need to develop more embodied communication in robots, not limited in verbal communication (Paradeda et al., 2016; Salichs et al., 2016; Matarić, 2017). Haptic sensing and feedback should be strongly considered in future research as part of multimodal perception and feedback (Arnold and Scheutz, 2017; Cangelosi and Invitto, 2017). More specifically, we need to implement the strategy to enable the robot to associate cognitive assistance and exercise with appropriate multimodal feedback, e.g., spoken words, facial expressions, eye gaze, and other body movements (Paletta et al., 2018). The embodied communication during human-robot interaction is a challenging research area (Nunez et al., 2015). It is still unclear how and how much the embodied communication from the robot can influence user's perception of the robot (Dubois et al., 2016). Moreover, previous studies indicated that users' experience of the robot could also be influenced by the unexpected behaviors (Lemaignan et al., 2015), synchrony and reciprocity (Lorenz et al., 2016), and even cognitive biases (Biswas and Murray, 2016, 2017) from the robot. A caveat is that there still exist many unknowns for natural human-robot interaction.

In summary, to achieve natural human-robot interaction during cognitive training requires not only multimodal sensing technology and artificial intelligence (e.g., deep learning) (Jing et al., 2015; Lopez-Samaniego and Garcia-Zapirain, 2016; Pierson and Gashler, 2017) but also the development of related fields (Wan et al., 2020), such as cognitive computing (Chen et al., 2018), social-cognitive mechanisms (Wiltshire et al., 2017), and modeling of cognitive architectures (Kotseruba et al., 2016; Woo et al., 2017).

## 5. Conclusion

Robot-assisted cognitive training is becoming an affordable promise for people with cognitive disabilities. In this review paper, we present a systematic review on the current application, enabling technologies, and main commercial robots in the field of robot-assisted cognitive training. Many studies have been successfully conducted to evaluate the feasibility, safety, usability, and effectiveness of robotic rehabilitation. Existing studies often include a small sample size. Also, the questionnaires need to be standardized both to evaluate the overall experience with the robot and the impact of the robot on the specific cognitive ability that it aims to assess. There are still multifaceted challenges in the application of robots in cognitive training. Firstly, ethical issues, such as human safety and violation of social norms, can arise during robot-assisted cognitive training. Secondly, with respect to the design of a robot-assisted cognitive training system, the developers should have a close collaboration with the end-users and stakeholders from the initial design, implementation, evaluation and improvement. Thirdly, the trust, reliability and the cost-effectiveness should be taken into account. Moreover, the rehabilitation robot should be capable to adapt and personalize to the specific individual need, and also learn to collaborate with users in the future. The recent advancement of social cognition may facilitate the human-robot interaction during cognitive training. Lastly, the rehabilitation robot should be able to interact with users in a natural way, similar to the human-human interaction during cognitive training. Noticeably, these challenges are mutually influencing one another. Cross-disciplinary collaboration is necessary to solve these challenges in future.

## Data Availability Statement

The original contributions presented in the study are included in the article/supplementary material, further inquiries can be directed to the corresponding author/s.

## Author Contributions

FY, RL, and XZ determined the review scope and review strategies. FY, EK, and ZL conducted the searching and screening of the literature, and reviewing of the selected articles. FY, EK, RL, and XZ wrote the manuscript. All authors contributed to the article and approved the submitted version.

## Conflict of Interest

The authors declare that the research was conducted in the absence of any commercial or financial relationships that could be construed as a potential conflict of interest.
